# Foreign

**Published:** 1841-10-02

**Authors:** 


### FOREIGN.

*Observations on the use of the Oxide of Silver, with Cases.* By C. H. B. LANE, M.R.C.S.L., L.A.C.—In the "Medico-Chirurgical Review" of last July, I first introduced the oxide of silver to the notice of the profession, I am now desirous of calling attention to the satisfactory results of more extended experience in its internal administration, which have fully equalled my expectations.

The consideration of the relationship of the bichloride and oxide of mercury first drew my attention to the analogy of the nitrate and oxide of silver. There was abundant evidence of the efficaciousness of the nitrate of silver as an internal remedy—an efficaciousness independent of causticity, which indeed, rendered it an uncertain and dangerous remedy. Looking at the analogy in question, it seemed probable that the oxide would prove a mild and manageable preparation of silver, bearing the same relation to the nitrate that the oxide of mercury does to the bichloride. The irritation of causticity must often interfere with this intrinsic action of silver; and this being avoided by the substitution of the oxide, is in itself a considerable advantage, as it often precludes the remedy being used as freely as is desirable. In cases where a lengthened administration of nitrate of silver is required—in epilepsy, for example—cutaneous discolouration has always been a matter of dread. Now, by the substitution of the oxide, this great objection will, I expect, be obviated; and with this view, the nitrate of silver, on being taken into the stomach, is converted into a chloride by the free hydrochloric acid of the gastric juice; this is taken up into the circulation, and when conveyed to the cutaneous surface is converted into an oxide by the action of light, and the strong affinity of albumen. This oxide cannot, apparently, permeate the capillaries, but becomes indelibly fixed, occasioning extreme disfigurement; but if the chemical process I have described be anticipated, and the silver is primarily introduced into the stomach as an oxide, its transmission to the skin would probably be prevented; for since the cutaneous capillaries are not permeable to its egress, when once deposited in the skin, as is the bile in jaundice, neither should we expect them to be permeable for its ingress. Of this view I have hitherto found no reason to doubt the correctness.

The caustic action of nitrate of silver, in itself highly valuable, is apart from our present consideration. What is the essential action of silver on the nervous system, the first medium of vitality, the great cincture of animal organization? I deem it primarily sedative, acting directly on the nervous fibre. Its speedy, often instantaneous effect, renders improbable that it acts through the medium of the blood, or by increasing the tone of the nervous system, and thereby allaying the irritability which is so generally co-existent with a state of asthenia. I believe silver exerts a peculiar sedative influence on the nerves of organic life, which control the capillary circulation, and through which the impulse to normal or abnormal secretion is conveyed. Thus it abates peculiar kinds of excitement, which exert an exhausting influence on the constitution; and the arrest of this process will doubtless constitute a secondary stimulant, or tonic influence, by turning nervous energy into its proper channels. The primary action of lead, on the other hand, appears to be on the nerves of animal life—those of sensation and voluntary motion; and I do not believe it demonstrates its antihæmorrhagic powers until it affects the composition of the blood, whereas the silver does so immediately on its administration, the result often becoming apparent with marvellous rapidity. The uterine system appears peculiarly obnoxious to the medicinal action of silver, and though most especially when there is undue secretion, yet its influence is not by any means confined to that morbid state alone. Whenever the uterine system is the centre of irritation, the oxide of silver will always produce more or less benefit. This position many of the cases I shall relate will support, though I do not by any means recommend its use in all cases of uterine irritation to the exclusion of other remedies: in many such cases, indeed, I should incline to prefer very different curative measures.

How, then, does silver act in epilepsy? Probably by the exertion of a sedative influence on the great nervous centre, analogous to that on the organic nervous system. It is only when the disease is idiopathic, however, that it can avail; when there is any organic change we may not expect much benefit; and it is so often the case that there is some structural lesion, that I cannot consider its effect in epileptic cases as any criterion of the good effect of oxide of silver; nor, indeed, have I had much opportunity of administering it therein. Two cases which I had for some time under my care were considerably benefitted; but in consequence of leaving town, I unfortunately lost sight of them. Dr. Golding Bird informs me that he has experienced great assistance from its use in some cases.

I have now repeatedly administered the oxide of silver during periods of more than two months without the slightest tendency to discolouration, even though in one case repeated salivation was caused, during which state the patient was seen by Dr. J. Johnson. Dr. Golding Bird, to whom I am much indebted for information on the subject, tells me that he has given it for four months without any appearance of discolouration. He has in three instances seen the gums affected during the use of the remedy, but otherwise has never found the slightest inconvenience experienced in more than a hundred cases in which he has prescribed it; he terms it a sedative tonic.

I shall now proceed briefly to recount the different cases wherein it has been administered, which have come under my observation within the last few months under their repective varieties, endeavouring to designate the morbid elements to which the remedy appears peculiarly opposed. All the cases have come more or less under the notice of my frieud, Mr. Dennett, he having treated them conjointly with me, and kindly afforded every facility for the illustration of my views.

*Case* 1. A. W., ætat. 45, had suffered with cardialgia and pyrosis almost constantly for nearly twelve years, scarcely a day passing without one or more attacks, so that her general health was considerably affected. The use of the oxide of silver in half-grain doses twice a day afforded complete relief.

*Case* 2. Mrs. R., ætat. 48; at the termination of a severe attack of intestinal spasm, nausea and pyrosis remained, which were quickly and permanently relieved by the remedy administered as before.

*Case* 3. W. B., ætat. 45, was under treatment for severe constitutional derangement, of which gastrodynia was at one time a prominent symptom, and was efficiently counteracted with the oxide of silver.

*Case* 4. Mrs. J., ætat. 30, was readily relieved of an irritable state of stomach, especially gnawing pain and nausea, by the use of the medicine for ten days.

*Case* 5. A. E., ætat. 26, had become much emaciated and debilitated: pulse quick and weak, and countenance exceedingly anxious. There was constant tenderness and sense of gnawing in the region of the stomach, and paroxysms of pain occurred once or more daily, terminating in the ejection of a quantity of clear water of saltish taste. There was considerable loathing of food; and when she did take it, a sensation of weight and fulness was induced; bowels regular; tongue with a red streak in the centre. Various remedies were used during a fortnight without avail, when the oxide of silver was resorted to, and its administration for ten days, to the extent of a grain daily, afforded effectual relief, though she had previously suffered five years. Since the above was written, she has had a slight recurrence of her complaint, after an interval of six months, which, however, is readily yielding to the same remedy.

*Case* 6. R. E., ætat. 30, was suffering with symptoms of gastrodynia, with much mental depression and constitutional debility. There was severe pain in the umbilical region, and left hypochondrium much aggravated by deep inspiration or pressure; tongue and pulse natural. Calomel and opium, bismuth, and various stomachics and aperients, were ineffectually tried during five weeks. A fortnight's use of the oxide of silver, however, enabled the man to resume his occupation as a labourer. After the lapse of about three months the complaint returned, and was similarly relieved.

*Case* 7. C. B., ætat. 40, within two months had lost his appetite, and become much debilitated. There was constant tenderness in the epigastrium, and paroxysmal attacks of pain several times a day, terminating in the ejection of a quantity of clear, tasteless water. The tongue was clean and natural, and the bowels free. Half grain doses of oxide of silver twice a day completely relieved the gastric symptoms in a week, but to remove the debility vegetable tonics were requisite.

*Case* 8. Miss S., ætat. 23, had for years suffered with severe gastrodynia, especially in the summer months, which numerous medical men had failed to relieve. Tenderness in the epigastrium, nausea, sense of distention and severe pain on taking food, which is often rejected, are the most prominent symptoms; much constitutional debility and irritation had resulted. The silver was administered in the usual doses with very immediate benefit, which has hitherto persisted.

*Case* 9. E. V., ætat. 31, had been suffering for five weeks with most distressing gastrodynia, which quite prevented his following his occupation as a blacksmith, and for which various treatment had been resorted to without avail. His state of mental depression was extreme; and though previously healthy and powerfully muscular, his strength was completely prostrated. He complained of the occurrence of violent pain at intervals in the region of the stomach; there was utter loathing of food, nausea, and occasional pyrosis; the epigastrium was very tender; the pulse and tongue were natural, and the bowels regular. Half-grain doses of oxide of silver three times a day speedily afforded relief, and in a fortnight he was able to return to his work.

In the above cases we find the oxide of silver subduing states of excitement and irritation, chiefly indicated by watery eructations, intermittent pain, and a sense of uneasiness and nausea, on which constitutional symptoms had supervened. The beneficial result was generally rapidly produced, reducing the morbid excitement, and restoring innervation to its normal channels. In cases where I deem organic mischief to have resulted, where the tongue is tumid and rather cracked, with a creamy surface, where there is *constant* gastric uneasiness greatly augmented on food being taken, especially if it be improper in its nature; the pulse weak and sharp, though often slow: in such cases the remedy is totally inefficacious. Dr. Bird informs me that in cases characterised by a glairy discharge from the stomach, which he considers analogous to the follicular gastric dyspepsia of Dr. Todd, he has completely failed in at least thirty cases in producing the slightest benefit by the use of the oxide of silver; and in those cases of pyrosis where I have found benefit, the discharge was certainly by no means glairy or viscid. He has seen the happiest results from the use of the remedy in gastrodynia, regarding it as a symptom of irritative dyspepsia.

*Case* 10. W. T., ætat., 32, has been out of health for some months, but had become much worse during the last fortnight. He had been daily more or less affected with dysenteric diarrhœa, passing at times a quantity of florid blood. Paroxysms of violent pain were frequently experienced in the epigastrium, after which a quantity of clear water of bitter taste was vomited. He had lost all appetite; pulse very weak, and much general debility. He was ordered to take half a grain of oxide of silver twice a day. Within three days the pyrosis was completely relieved; the diarrhœa was much restrained, hardly any blood being passed, but diuresis was very troublesome for a few nights. He remained, however, in a deplorable state of weakness, which required an assiduous administration of tonics and nourishment. The pyrosis has never returned. The diarrhœa recurred from time to time, but without hæmorrhage; and was always readily checked by two or three oxide of silver pills, though unaffected by opium.

*Case* 11. J. S., ætat. 47, had been ailing a fortnight, eight or nine evacuations from the bowels taking place daily without much pain. They were fluid, dark, and often mixed with blood. The abdomen was tender, especially in the site of the colon; pulse 80, weak; tongue red, and somewhat glazed. Eight doses of oxide of silver at intervals of six hours completely cured the man.

*Case* 12. Mrs. C., ætat. 40, remained in a state of great debility after fever, and an intractable diarrhœa came on, twelve or more evacuations occurring daily, with great pain but no hæmorrhage. For a fortnight all the usual means were tried without the diarrhœa being effectually restrained, when the oxide of silver was resorted to, and complete success attended its administration in the course of a few days.

*Case* 13. Miss J., ætat. 24, was subject to periodical attacks of diarrhœa. One more severe than usual was readily arrested by oxide of silver, but at the same time menstruation was completely checked for twenty-four hours, occasioning some inconvenience.

*Case* 14. Mrs. V., ætat. 50, was greatly relieved from chronic diarrhœa by a week's administration of the oxide of silver.

*Case* 15. Mrs. P., ætat. 30, was temporarily relieved by oxide of silver, of periodic diarrhœa, but the complaint soon recurred; and as it was found to depend on an antonic state of the digestive apparatus, the food being passed in an unassimilated state, iodide of iron was substituted with complete success.

In troublesome cases of idiopathic diarrhœa or dysentery, attended even with much irritation, the effect of the oxide of silver was strongly marked, and its sedative action clearly distinguished. Beyond checking an exhausting secretion, it did not appear to exert the slightest tonicity; it always being requisite, where debility existed, to resort subsequently to corroborant treatment. In Case 15 the respective action of oxide of silver, and ioduret of iron were well contra-distinguished. Also, in Case 13, the influence on the uterine function was remarkable.

*Case* 16. W. W., ætat. 32, a stout, healthy man, complained of excessive nocturnal sweats, without the slightest trace of any organic mischief of any kind. The use of the oxide of silver for a few nights was completely successful in checking the diaphoresis.

*Case* 17. P. K., ætat. 54, subsequently to a slight febrile attack, became subject to excessive diuresis, having occasion to get out of bed three or four times in the night, when he would pass three or four pints of water. There was constipation, clammy mouth, and excessive appetite. After giving a dose or two of opening medicine, the oxide of silver was administered, and by persisting in its use for a fortnight the flow of urine was so completely abated, that the man never had occasion to get out of bed above once in the night. The other symptoms, also, quite disappeared.

These last two cases are instances of the arrest of excessive secretion by the oxide of silver. It was used in a case of mellituria, with only a slight temporary effect; but I could have wished that the use of the remedy had been pushed further. It was also used in a case of hematuria, and afforded some relief, but the copaiba was found far more efficient.

*Case* 18. L. J., ætat. 14, became affected with a slight menstrual show, and subsequently, probably in consequence of the uterine excitement, she suffered from violent pain in her legs, preventing her from getting about. A week's administrstion of the oxide of silver afforded complete relief.

*Case* 19. C. C., ætat. 27, had long suffered from constant ill health, with severe dysmenorrhæa and various neuralgic symptoms. No remedy afforded her permanent relief, but the oxide of silver made her feel so much more comfortable, that when removed from the neighbourhood she sent a considerable distance to obtain a supply. It doubtless had some influence over the uterine irritability.

*Case* 20. E. M., ætat. 26, had been for some years affected with uterine disease. The use of the oxide of silver had an obvious effect in diminishing pain in the region of the uterus, and diminishing the muco-purulent discharge.

*Case* 21. M. P., ætat. 21, was suffering with pulmonary abscesses, unconnected with phthisis. There was great nervous irritation, and violent pain in the epigastrium, which were much relieved by the oxide of silver. The pulse gradually fell in a fortnight from 110 to 92. Powerful counter-irritation and tonics were subsequently successfully resorted to.

The above four cases are calculated to bear out the opinion of the sedative influence of silver.

*Case* 22. Mrs. M., ætat. 35, had been suffering from severe menorrhagia for a fortnight, which was controlled in twenty-four hours by oxide of silver.

*Case* 23. Mrs. M., ætat. 46, had menstruated several consecutive times to excess, attended with much pain; the present attack was much more severe, so that she was compelled to seek medical relief. The flux had lasted more than a week, and, instead of abating, was on the increase. The discharge came in large clots, with violent pain, much increased on the slightest motion. It was greatly abated by three doses of the oxide of silver, and in the course of three or four days the discharge was quite arrested, and the pain completely subsided. During four subsequent months she experienced no particular inconvenience at the monthly periods; at the fifth her complaint recurred with violence, but was subdued with facility by resorting to the same treatment at an earlier period. Slight giddiness was occasioned, which was immediately relieved by an aperient.

*Case* 24. Mrs. M., ætat. 45, had suffered for some months with leucorrhœa, and also menorrhagia, but at the time of application was only suffering with the first complaint. She had much pain in the back and down the thighs; the discharge was profuse, and there was great general debility. Oxide of silver, administered in the usual way, arrested the leucorrhœa, and when she did menstruate the flux did not come on to excess at the two subsequent periods.

*Case* 25. Mrs. S., ætat. 34, had been out of health a year and a half, in consequence of a severe mental shock; profuse menorrhagia coming on at short and uncertain intervals. There was also abundant leucorrhœa. These affections had produced pain in the back and side, and great debility. The oxide of silver was given in half grain doses twice a day, and at first disagreed somewhat with the stomach and bowels, but within five days the leucorrhœa was stopped, the pain relieved, and she consequently felt much stronger and better. The next menstrual period came on after a much longer interval, and more moderately than for some time previous. The amendment has hitherto persisted.

*Case* 26. Mrs. A., ætat. 31, became affected with a considerable sanguineous discharge, when between four and five months advanced in pregnancy. It was unattended with pain, and had gone on for some days previously to her applying for advice, though without occasioning any particular weakness. Six doses of oxide of silver, at intervals of six hours, quite restrained the flux.

*Case* 27. Mrs. M., ætat. 29, was about four months gone in pregnancy when violent flooding came on; this was restrained for a few hours with the oxide of silver, when the hæmorrhage recurred in an alarming manner; and the placenta being found attached over the os uteri, further interference became requisite to effect immediate delivery.

*Case* 28. Mrs. T., ætat. 40, had long been affected with chronic enlargement of the womb, and had been subject to violent flooding from time to time at the menstrual periods. She had this time been affected more than a week, the discharge being abundant and clotted, and attended with much pain in the abdomen and back. It had caused much general debility, and the bowels were rather relaxed. She had suffered severely some time previously from an injudicious attempt to restrain the discharge with acetate of lead and opium, which occasioned a severe illness, and inspired her with dread of any attempt at arresting the flow. Half a grain of the oxide of silver was ordered twice a day, and the best effects were soon manifest from its administration. In forty-eight hours the flux was completely restrained, without the slightest ill effect, and she was quite freed from pain. The next menstruation occurred in moderation.

*Case* 29. Mrs. B., æt. 42, was suffering with excessive menstruation, with the usual symptoms. It was readily restrained in twenty-four hours with the remedy.

*Case* 30. Mrs. S., ætat. 34, was affected with severe and alarming menorrhagia, which was checked with difficulty by sulphuric acid and opium, leaving her excessively debilitated. Two months subsequently the complaint recurred with equal violence, but was far more readily arrested by eight doses of oxide of silver; nor has it recurred after the lapse of five months.

*Case* 31. Mrs. R., ætat. 36, had been labouring under excessive menorrhagia for a fortnight, the discharge being clotted and attended with much pain. The complaint had reduced her greatly, and was getting worse daily, so that she was scarcely able to sit up in her chair. By the use of the medicine the complaint was completely abated in eight-and-forty hours, but it required a fortnight's tonic treatment to restore her strength.

This last series of cases is as valuable as it is remarkable, and the *invariable* control exerted over uterine fluxes was as gratifying as unexpected: together with some of the cases previously narrated, the results appear strongly corroborative of my opinion, that silver exerts a peculiar medicinal influence over the uterus. In Cases 26 and 27, I certainly did not anticipate any effect when a trial of the oxide of silver was suggested by my friend, Mr. Dennett. The action of silver is very widely different from the astringent action of lead, or even sulphuric acid: and the contrast is well exemplified in Cases 28 and 30. I have never seen febrile excitement result from its administration in any one of the above cases, such as we see occur from the common astringent remedies, and the only approach to inconvenience was in Cases 23 and 25. In the first of which the head suffered somewhat on the menstrual cheek; and in the second, on the first administration of the medicine, the stomach and bowels were somewhat upset. In those cases where leucorrhœa was benefitted, I consider it uterine, from the connection it had with menorrhagia, and as I have repeatedly found no influence exerted over mere vaginal discharge. Dr. Golding Bird likewise entertains a favourable opinion of the oxide of silver in menorrhagia.

I may here mention a curious fact, which was related to me by a friend. He had ordered the oxide of silver for two ladies suffering with uterine irritation and menorrhagia with apparent benefit; neither of them had borne a child for some years, but immediately after being thus relieved both became *enceinte*. Thus an abnormal excitement being abated, the womb became susceptible of its natural stimulation, even as relief of irritability of the stomach will afford tonicity to the natural digestive function.

In conclusion, I beg to state that I do not by any means claim any specific action for the oxide of silver, though I do consider the influence exerted by it as very peculiar. It has been my object to specify and individualise that peculiar influence in order that due value might be assigned to it, so that it might be available, and its effect recognizable, in complicated disease. In all the above cases I have been most careful to administer the remedy simply, without the complication or interference of any other medicine.

I have found considerable difference in various samples, some being exceedingly irritant, and even caustic, probably from having a compound salt in combination. The most obvious criterion of the goodness of the preparation is the colour, which should be an olive brown, and not, as I have frequently seen it, nearly approaching to a black. The light and air should always be carefully excluded.—*London Lancet.*

---

*Observations on the use of the Vectis, or Single-Blade Extractor, in difficult labours.* By James Borrett, M. D., Physician to the Dispensary, and Accoucheur to the Lying-in Charity in Norwich.—I propose in this communication to consider the claims of the vectis as a safe and efficient mechanical power, in accomplishing difficult labours in a manner similar to nature, by supplying and increasing uterine contraction.

As the following observations are intended to be altogether practical, I abstain from entering upon the early history and introduction of the vectis into midwifery practice, referring my readers to Dr. Bland's learned historical memoir of that instrument,* published in the second volume of the London Medical Communications, in 1790.

Dr. Bland, in that paper, while stating the comparative utility of the vectis and forceps, gives a striking instance of the successful application of the former, related to him by Dr. Garthshore, by which "Dr. Bromfield, whose prejudices against the vectis were as strong as even those of Dr. Osborne, was compelled to acknowledge he had been mistaken in his opinion." Strongly impressed with the superior utility of the vectis, Dr. Bland used it on the principle of a lever of the first kind. Still he makes mention of Mr. Dease's "Observations," published in 1783, to whom the profession is indebted for its appropriate appellation and use as an *extractor*; indeed, until the time of Mr. Dease, the vectis was made with so slight a curve in the blade as to render its abuse as a lever inevitable. Such an objection cannot be urged against Dr. Lowder's vectis, which, by its construction, is admirably adapted to aid as an extractor in lingering and impeded labours, as its introduction and application on the principle proposed by Mr. Dease, was a decided improvement in the state of instrumental delivery practised at the same time.

Dr. Denman likewise expressed himself in terms so commendatory of the vectis, that Dr. Osborne, who lectured with him, not choosing to be considered as having adopted his opinion, and fearing "it would tend to establish the preference of the vectis," takes exceptions to the following paragraph in Dr. Denman's comparison of the vectis with the forceps. "That the vectis, prudently used, is, in every case, an equally safe and efficacious instrument with the forceps, and a better adapted instrument in many cases, which occur in practice."

In 1794, Dr. James Hamilton published an admirable essay on the use of Lowder's vectis, in which he has stated in a clear, correct, and convincing argument, its utility as a mechanical expedient in the second class of laborious labours.

I had not seen Dr. J. Hamilton's essay till after I had written the greater part of these remarks; and as I found we agreed in our views, I was not surprised in finding also a corresponding agreement in much of our language. Dr. J. Hamilton's opinion of the vectis, however, changed in the course of a few years; for we read in his "Practical Observations," the reasons which for many years have induced him to discountenance the use of that instrument, with some exceptions; "having found," as he states, "that by limiting the first stage of labour to twelve hours' duration, laborious labours very rarely occur in his own practice," and that when they do, he gives a preference to the forceps.

To those accoucheurs who do not approve of Dr. J. Hamilton's active interference in the first stage of labour, his reason for relinquishing the vectis does not apply; but admitting, that by venesection and the opiate enema,* by support and counter pressure, and by sliding up a portion of the cervix uteri compressed between the infant's head and the bones of the pelvis, the accoucheur renders the complete dilatation of the os uteri less tedious, and thereby the termination of the labour by the natural powers more frequent—admitting, according to many authorities, that the absolute necessity of applying either the forceps or vectis is very rare—nevertheless I am satisfied that the vectis can be used as an extractor in many cases with a certainty of diminishing the duration and severity of the labour, and therefore with advantage to the mother, and with a better chance of saving the life of her infant. Moreover, I am not disposed to concur in the exclusive preference given to the forceps by Dr. J. Hamilton, in those laborious labours which he describes as rarely occurring in his practice, any more than in the cases wherein accoucheurs commonly apply the forceps, because from my own experience I have found the vectis all-sufficient. Although the authority of Dr. J. Hamilton for the use of the vectis be withdrawn in accordance with his own practical views, his argument in its favour holds good, and will have its due weight with the profession. To such high authorities as Drs. Denman and Hamilton, I may add those of Lowder, Haighton, Blundell, Breen, Gaitskill,† and the writer in the Medico-Chirurgical Review, July 1, 1821-22. Passing over the many eminent men who, advocating the superiority of the vectis, used it altogether as a *lever*,—Dr. Blundell, in his Lectures, says, "the lever is of more importance to the general practitioner than either the long or short forceps, and I more particularly recommend it to you."

I would here take leave to observe that Dr. Collins' doctrine of‡ "waiting till the patient's strength be much exhausted," or, "so long as

* Observations on the Obstetric Extractor, by Dr. Breen. A valuable paper published in the Dublin Journal of Medical and Chemical Science, July 1, 1835. See also Principles and Practice of Obstetric Medicine and Surgery, by F. H. Ramsbotham, M. D.

* Of the opiate enema I am unable to speak from my own experience.

† See London Med. Repository, vol. 20, 1823.

‡ Consult the paragraph. Dr. James Hamilton's Comments and Dr. Collins' rejoinder are before the profession.

the head advances ever so slowly," is not, in my opinion, the right practical rule for the guidance of the accoucheur in the use of either forceps or vectis. Such a doctrine is very like instituting an experimental observation as to the degree and duration of suffering a parturient woman can endure, with a bare chance of escaping injury both to herself and infant. Dr. Collins' imperfect estimate of the real value of the forceps is only, I conceive, to be explained by the faulty dimensions of the instruments he describes. Hamilton, Burns, Merriman, Naegele, Osiander, Siebold, advocate juster views of the advantages of the forceps. In my own practice, adopting in general Dr. James Hamilton's management of the first stage of labour, I use the vectis not only in those rare cases in which he applies the forceps, but also in various cases in which, if not absolutely required, it may yet be advantageously employed.

Much has been written on the comparative merits of the vectis and forceps, but until the time of Dease and Lowder, the main objection against the abuse of the former had not been completely met, nor the claims of the vectis or *single-blade extractor*, and of the forceps or *double-blade extractor*, by teachers and practitioners, fairly estimated. Since that time, the vectis has been used by experienced and successsful accoucheurs, not only in cases wherein the duration and difficulty of the labour justify its application by every right precept and rule of practice, but in those cases also in which the most procrastinating practitioner has a reluctant recourse to the forceps.

It is, indeed, singular, but not less true, that what ought to have been deemed a ground of preference of the vectis, namely, the greater facility of its application, has, by the almost unanimous voice of lecturers, been pronounced as a conclusive objection to its general introduction into practice. Such, indeed, might have been held to be a good reason against its *abuse* as a lever in the hands of coarse, rude, and bold pretenders, but in these days of advanced and diffused knowledge, and of higher qualifications required by licensing bodies, such an argument can fairly have no weight. But if there be those who still entertain fears of the recurrence of such grievous cases as have been related by Dr. Merriman and Mr. Gaitskill, I would suggest a coroner's inquest as a summary remedy for such instrumental "malapraxis."

On no ground can a meddlesome midwifery be defended, which uselessly interferes with, or injuriously interrupts natural labour. On the other hand, humanity calls for, and reason and experience support that line of practical duty, by which anxiety of mind and bodily suffering are abridged without the least risk to the parturient woman.

The advocates of the vectis have been twitted for using an instrument which has not the power of diminishing the child's head, nor indeed any great mechanical power at all, when simply applied on the principle of an extractor. I shall endeavour to show that this opinion, which I believe to have led teachers to discountenance the use of the vectis, is both erroneous and absurd.

It seems to me, that teachers, by a plausible exposition of the application of the forceps, however ingeniously correct, as applied to the dried fœtal cranium upon the lecturer's table, yet of utter practical fallacy, when used to help an infant's head through the bony canal of the pelvis, have unfairly prejudiced the minds of rising practitioners in favour of that instrument.

When the lecturer exhibits to his class the dried cranium, and explains the advantage of one forceps over another by reason of its fenestræ admitting the parietal eminences, or by the inclination of one blade permitting a more exact adaptation of surfaces; when he insists, that the forceps, not projecting beyond the most salient points of the head, does not therefore encroach upon the pelvic cavity, he is surely inculcating a great practical absurdity, which observation and reflection should correct. According to my view, the vectis and forceps occupy space in the pelvis just in proportion to their bulk: and consequently the *forceps, with two blades*, adds to the bulk which must pass through the bony canal twice as much as the *vectis, with a single blade.*

By an admirable provision of nature the fœtal head is so made up of component portions freely moving upon each other, that it is capable of assuming almost any form required to traverse the unyielding bony channel through which it is forced by the "vis à tergo" of the contracting and propelling uterus. What accoucheur has not felt the semi-membranous, semi-osseous, multipartite head squeezed through the irregular form of an abnormal outlet, moulded accurately to it, and presenting no resemblance to the symmetrical conformation of the dried cranium upon the lecturer's table?

To suppose that in a narrow and deformed pelvis the head preserves its natural form under the gripe and pressure of the forceps, as when its application was exhibited to us in the class, is clearly an untenable proposition, which our experience soon disproves.

Taking for example a case wherein the natural powers fail to expel the infant: the question to be solved is, what means can most easily bring the compressible head through the pelvis, to whose form it can be moulded? I answer, such a mechanical power as, occupying the least bulk, will accomplish its delivery. Now, as the single-blade extractor or vectis occupies but half the space of the forceps or double-blade extractor, the vectis is certainly that mechanical expedient. The forceps, being an instrument of still greater power, may have in certain cases *pro tanto* a superiority, but as the vectis is commonly all-sufficient, almost superseding a necessity for the forceps, the vectis has assuredly the first claim upon the accoucheur. It has besides many advantages; it is easy of application, makes only a harmless interrupted pressure on the child's head, and does not act at all upon the structures of the mother. With these combined advantages, "it affords," to use Dr. Hamilton's words, "exactly the assistance in the second order of laborious labours which is required. For as the size of the child's head is in natural cases diminished as far as is necessary, by the contractions of the uterus forcing it forward through the bones of the pelvis, an increase of the vis à tergo will of course increase that diminution, if the shape of the passage require it. While Lowder's vectis, therefore, possesses the power of compressing the cranium in common with the forceps, it has a decided superiority over them in this, that it accomplishes that end by similar means with nature."

In commencing midwifery practice I accustomed myself to the use both of the forceps and vectis, but after a time became so strongly impressed with the applicability, safety, and efficiency of Lowder's vectis used as an *extractor*, and more especially satisfied with the rationale of its action, either supplying the place of the propelling power or increasing their efficacy, that I have in a great measure relinquished the forceps. I do not, however, advocate an exclusive use of either instrument.

To Lowder's I give the preference, and likewise find it more convenient for the pocket.* I have often used the vectis without the knowledge of the patient or her attendants: sometimes (which is perhaps the better way) I have explained its use to the patient, who in a subsequent lingering labour has urged me to have recourse to it. The vectis is to be applied in the following manner:—

The woman lying on her left side at the edge of the bed, with the knees drawn up and feet resting against the bed-post or back of an attendant—a round towel, in cases of difficulty, being fixed to the same bed-post, for a purchase during each labour throe—the lower bowel and bladder being ascertained to be empty, the os uteri dilated, and the Liq. Amnii discharged, the instrument being warmed by putting it up the coat sleeve with the blade resting on the palm of the hand, and greased, if required by deficient vaginal moisture, two forefingers of the left hand are to be passed along the vagina towards the sacral promontory, or to the posterior lip of the uterus, if it can be felt, and the vectis guided by them is to be carried within the os uteri, being kept closely applied to the head by the curve of its blade: its direction is then to be changed by a sliding-movement to the left or right sacro-iliac synchondrosis; it is next to be carried upwards, and by a combined sliding and slight semirotatory motion brought round to the hollow of the ilium, or ramus pubis, and fixed over the occiput; or the vectis may be made to describe a semicircular movement, the fingers of the left or right hand serving as a fulcrum, on which it turns by the help of the other hand. When it is felt that the instrument has a firm purchase, an extractive effort is to be made during a pain in the axis of the inlet, the practitioner resting when the pain goes off, and thus alternately acting and resting in imitation of natural pains. If the head is detained so high up that by passing the hand within the vagina its position cannot be ascertained, the vectis is to be directed to the hollow of the left ilium, as the first position is by far the most frequent. According to a statement in the "Journal de Medicine," given by Dr. Merriman, the first position occurs in five or six cases where the second is once found: according to Baudelocque the first and second positions occur in the proportion of 7 or 8 to 1. Naegele, however, in his "Mechanism of Parturition," states the *third** position to be next in frequency after the first; the *third* occurring in proportion to the first as 1 to 2½.

If, for example, the head is detained at the inlet, by reason of the head and inlet not having a correlative proportion, if the head does not enter in its right axis, or, if only one-third part of it has passed the brim, that movement of flexion upon the chest, which traction upon the occiput operates, will give the direction in which Capuron describes the head entering the pelvis, namely, with the chin approximated to the chest; the vertex or smallest part of the head being in the direction of the largest diameter of the pelvis. I have known my father to bring down the head by the vectis, when it could only be touched by the hand passed per vaginam. In this case, the practitioner had been foiled in the application of the long forceps, and as the liq. amnii was discharged, turning could not without difficulty have been accomplished.

When the head has entered the cavity of the pelvis, and the accoucheur has ascertained its position by the anterior fontanelle sutures, or the ear, the vectis, passed as before along the sacrum, is to be carried round either below to the left or above to the right ramus pubis, according as the face is turned to the left or right side, and applied over the ear, face, and chin. We commonly find the head engaged transversely in the cavity, and have only to determine to which side the face looks.

If the head occupy the fourth or fifth posi-

* This observation exposes me, I am aware, to the risk of being numbered amongst the proscribed who go to no labours without instruments in their pockets.

* The *third* position of the German schools corresponds with the *fourth* of Baudelocque.

tions of Baudelocque, with the anterior fontanelle to the left or right acetabulum, we shall expect to find that the uterine contractions carry the face* into the hollow of the ilium or to the sacro-iliac synchondrosis, and the occiput in a corresponding opposite direction. If the natural pains do not effect this movement, we may succeed in bringing with the vectis the occiput to the acetabulum where it is found in the first and second positions: should we fail to accomplish this, the head having descended too low down, the chin may be brought under the arch of the pubes. After allowing due time for the expansion of the perineum and moulding of the head, if the uterine action become feeble and inadequate to the delivery, we are to apply the vectis over the occiput laterally as a single blade of the forceps, or posteriorly along the sacrum, and directing the head towards the pubis to deliver by a gradual extractive force, while with the left hand we support and carry forward the perineum and nates. By the vectis we can rectify the transverse malposition of the head, described by Dr. Montgomery, in which the labour throes follow quickly and powerfully without in the least degree advancing the head. After we have rectified a malposition of the head, or a forehead or face presentation, or brought the arrested head through a narrow inlet, we should withdraw the instrument, and leave the case to be terminated by the natural efforts.

The accoucheur will find that, in numerous cases, he can terminate by the vectis, with advantage to his patient, a lingering or laborious and exhausting labour, in which one practitioner would have recourse to ergot, another to laudanum, and a third, as Gosch quaintly expresses it, to *tincture of time*.

Two rules are to be constantly observed in the use of the vectis:—Firstly, to co-operate with each contraction of the uterus, resting always in the intervals of the pains; secondly, to apply our extracting power in the axis of the pelvis, according as the head occupies its inlet cavity or outlet.

In the fourth clinical report of difficult cases in Midwifery, by Dr. R. Lee,† he observes that "had a faithful report been given of all the cases of artificial delivery contained in this table" (exhibiting a comparative view of instrumental delivery by the forceps, and craniotomy, in the several British and Foreign Hospitals,) "it is impossible that so great a discordance of opinions could have so long existed respecting the employment of instruments in the practice of midwifery."

With a view to aid in rightly appreciating the advantages, difficulties, and ruinous consequences resulting from the use of instruments in obstetric practice, I proceed to relate the following twenty-two cases, for the accuracy and fidelity of which I can vouch, wherein I employed the vectis. They present to the accoucheur practical data, which may be advantageously contrasted with the forty forceps cases recorded in the valuable series of clinical reports above mentioned. I am strongly inclined to the opinion, that a preference is due to the vectis in such cases, and that prudently used (and I entertain no fears of its abuse, certainly none which can be compared with the grievous injuries inflicted in numberless instances by the forceps,) it will be found worthy of more general employment by the accoucheur, and of the sanction and recommendation of midwifery teachers.

The nine cases in which I applied the forceps, present, it is true, no circumstances demonstrative of the comparative difficulty and danger in the employment of that instrument; still, in not a single case should I have failed, I believe, to accomplish the delivery with the vectis, and the favourable results are doubtless to be attributed to an earlier employment of the forceps than in the instructive cases recorded in Dr. Lee's report.—*London Med. Gaz.*

[*To be continued.*]

---

*Case of Rupture of the Intestine occurring during the taxis employed for the reduction of an Incarcerated Hernia.*—Elizabeth Rivett, ætat. 55, admitted into St. George's Hospital June 16, 1841, under the care of Dr. Wilson, with palpitation, loss of appetite, and other dyspeptic symptoms: these were relieved by hydrocyanic acid and mild tonics, and she was to have left the Hospital upon Wednesday, the 7th of July.

On Sunday, the 4th inst., at ten, A.M., she requested the house-surgeon, Mr. Lee, to reduce the left femoral hernia, which had resisted her own attempts during the previous hour. She was placed immediately in a warm bath, when, without any immoderate pressure, the bowel appeared to return with a gurgling noise into the abdomen. Not many minutes had elapsed before she began to complain of pain over the whole abdominal region; her face became flushed, and her pulse greatly accelerated, indicating about 90.

At two, P.M., Mr. Cæsar Hawkins saw her, and found a small hernia, about the size of a walnut, in the left femoral region, and also an umbilical hernia. When touched, neither appeared tense or more painful than the other part of the abdomen, which was acutely sensible to pressure. She complained of some retching and nausea, but had not brought anything off her stomach. Pulse 100, somewhat sharp. In order to ascertain for certain whether the bowel was or was not obstructed in the sac, she was ordered eight grains of calomel, to be

---

* We are indebted to Solayres, who followed Ould and Smellie in investigating the mechanism of labour, for this observation, which has been confirmed by Naegele and Baudelocque, Jun.

† Vide Lond. Med. Gaz. New Series, vol. 2, session 1838–39.

taken immediately, and an injection, containing an ounce of castor oil, in three pints of decoction of barley. Half an ounce of castor oil, with four drops of tincture of opium, to be administered two hours after the preceding remedies, if necessary.

Six, P.M. Only a small part of the injection had come away. She still suffers great pain over the whole abdomen. Pulse 120, more sharp. Bleeding to ten ounces, not buffed or cupped; calomel, two grains; powder of opium, one-sixth of a grain: to be taken every two hours.

Ten, P.M. Has taken two powders without a relief from the bowels; very restless and anxious; pulse reduced to 110, by the bleeding. Calomel, ten grains: to be taken immediately. Eighteen leeches to the abdomen.

July 5. The severity of the pain had entirely prevented sleep; countenance pallid, and bedewed with perspiration; the bowels have not yet been open; great pain and distention of the abdomen; the sac in the femoral region not tense, but apparently containing air; much worse.

One, P.M. At a consultation of the surgeons an operation was resolved upon, in order to examine the state of the bowel.

Operation, half-past one, P.M. Mr. Hawkins made a longitudinal incision over the tumour, and the hernial sac was exposed by dissection: this was found to be considerably thickened. A small opening was made in the sac, when about four ounces of peritoneal fluid with some air escaped. Nothing was found in it. A hernia-knife was now used to divide he seat of the stricture, and the finger introduced into the abdominal cavity; immediately, air and fæces followed their withdrawal. The case was now no longer doubtful; a piece of oiled lint was placed in the wound, and the patient left to her fate, or to the slight chance of recovering with an artificial anus after the operation. The symptoms not being relieved, died at eleven. P.M. Some fæces escaped from the wound.

*Post-mortem Appearances Thirteen Hours after Death.*—Body well developed.

*Thorax.*—Lungs perfectly healthy; pericardium and heart without any perceptible signs of disease.

*Abdomen.*—Upon opening this cavity, the parietal and visceral peritoneum was found to be very vascular. The convolutions of the intestines were glued together by recently-effused lymph. Posteriorly the intestines were in contact with a large quantity of fæcal fluid; and here a much greater degree of vascularity was observed than elsewhere. The fæcal fluid after minute examination, was found to have exuded out of a circular opening in the ileum: its edges were round, and an ulcer had evidently been formed some time previously to her death in this spot. The opening did not correspond, by an nch or two with the strictured portion of the sac. At the lower part of the abdomen lymph had been effused, so as to form a kind of pouch, in which fæcal fluid, the residue of that which escaped during the operation, was contained. The other organs were not examined. It is probable that if the ruptured portion of the intestine had remained, as is sometimes the case, in the sac, the operation might have been attended with more success; but as it was, the retraction of the intestine after the reduction caused a general diffusion of irritating matter, and thereby nature's efforts to confine the mischief were rendered useless.—*Ibid.*

---

*Tubal Pregnancy issuing successfully.*—A woman, thirty-two years of age, who had borne several children, in the month of May, 1834, suffered from vomiting, and complained of pains on the right side of the abdomen in the region of the ovary. In this situation she perceived a tumour, which was small at first, but in six weeks became as large as the head of a child. The pain extended along the back and the right thigh, and soon became so severe as to deprive her entirely of sleep. There was constipation, fever, thirst, and subsequently jaundice, with slight infiltration of the skin.

M. de Ritzen was called in, at about six weeks after the commencement of the pains. The woman believed herself to be pregnant, in consequence of the cessation of menstruation and from other signs.

The tumour was at this time exceedingly large; slight pressure increased the pain, but greater pressure caused it to cease. The right side of her person was more œdematous than the left; the right pupil was more contracted and smaller than the left, and the whole of the right side affected with a general sensation of uneasiness. The liver was normal, pulse quick, skin hot and dry, thirst considerable, tongue covered with a yellowish coating, appetite bad.

On examining the uterus through the vagina, the orifice was found towards the left side, the lower lip being enlarged. Through the cul-de-sac of the vagina on the right side he felt a tumour immediately above the uterus, of which he could easily feel the movement, when pressure was made in the right groin. On examining the tumour through the rectum, it was found to be a little larger in this situation. For three months no discharge had taken place through the vagina. Her bowels were at first opened every four days by means of clysters and saline purgatives; but at the end of twelve days there was complete constipation, and the transverse colon might be felt distended through the abdominal parietes.

By these signs it was presumed to be a case of tubal pregnancy, and auscultation rendered this diagnosis still more positive, discovering a bellows sound at the same instant with the pulsation of the arteries. The tumour now seemed likely to burst; a narcotic emollient poultice was therefore applied, and a seton made a little to one side. With the intention of promoting premature delivery, a dose of salts was prescribed, and subsequently pills composed of ergot of rye and extract of aloes. In twenty-four hours the pains in the tumour ceased, and on the third day the patient was able to sleep. The pills had at first produced vomiting, and subsequently contractions in the tumour and in the uterus; the patient expelling through the vagina some blood of a deep colour, and in twelve hours her bowels were moved. After this, large clots of blood were discharged, which were found to be composed of flocculent tissue like decayed membrane, and afterwards part of the chorion was found, but nothing which indicated the presence of an embryo.

On the fourth day the tumour had considerably diminished, but did not disappear altogether till after about a year and a half. During nine months she was subject to frequent discharges of blood. The bellows sound began to diminish immediately after the treatment, and disappeared completely in three weeks. The ergot of rye and aloes were administered for three days, and the Epsom salts during eight days: the seton was retained for several months. The woman is now well, and has not again become pregnant.—*Ibid.*

---

*On the use of Ergot.* By George Fife, M. D.—Having long been convinced of the value of the ergot of rye as a medicine, I now beg to offer the following remarks to my professional brethren; premising, that what I am about to advance is not the offspring of any fanciful notions as to its *possible* effects, but a simple statement of the results by which its use has been followed in my practice for several years. This assurance seems to be especially necessary at the present time, when it is but too much the fashion to render facts subservient to hypotheses. Whatever, then, may be advanced in this communication has been actually observed; and I am perfectly satisfied (due discrimination being exercised) will be fully sanctioned by further experience.

To enter upon the consideration of the action of the ergot on the parturient uterus, or the very interesting and important question of its power to excite uterine action, is foreign to my present object; I shall therefore proceed at once to a statement of its efficacy as a medicine, and the diseases in which I deem it worthy of confidence. They are--polypus uteri, attended with profuse hæmorrhage; menorrhagia, where there is no inordinate action of the heart or arteries, or morbid sensibility of the uterine system; in leucorrhœa, when independent of inflammatory action; in chlorosis with amenorrhœa; and in dysmenorrhœa: in all of which cases I have had numerous opportunities of ascertaining its efficiency.

The first time I saw it exhibited as a medicine was by my lamented friend, the late Mr. Parr, of this town, so long ago as the year 1828. It was in a case of very large polypus of the uterus, accompanied with frequent and frightful attacks of hæmorrhage. He gave the ergot after all ordinary treatment had proved unavailing. The effect produced was not only the moderation of the hæmorrhage, but also the expulsion of large and numerous masses of the tumour, in many of which a distinct fibrous structure was perceptible. After its continued employment, the woman, who had arrived at the climacteric period, enjoyed comparative health and comfort, being freed from the repeated and alarming hæmorrhages, and experiencing no inconvenience from the small portion of the tumour which, when last examined, still remained. To this case I owe the idea that it might possibly be useful in others. How far this has been justified, the sequel must decide.

In menorrhagia, by which term I do not mean the mere increased menstrual discharge, I have found this medicine of the greatest value. In this disease, however, it is necessary to ponder well on the individual circumstances of each case, and to use the utmost caution in ascertaining the cause on which the disease depends; as where such precaution has been neglected, it has been my lot to see both the sufferings and danger aggravated by the ergot. In this respect it does not differ from other active medicines. The slightest reflection will suffice to call to mind the very different states of the system in which this disease occurs. It may, for example, happen in the most phlogistic and plethoric, or it may arise in a person of a diametrically opposite constitution. If given in the first state it is decidedly prejudicial, unless preceded by such means as are calculated to remove alike the plethora and morbid sensibility; and even when this has been done, its operation requires to be carefully observed. In the last it acts most beneficially, as it at once raises the nervous energy of the uterus, and through this medium probably imparts increased tone to the relaxed and debilitated vessels from which the exhalation takes place. The following case affords a striking example of the utility of the medicine:—

In 1834 I was requested to visit a poor woman who had for many years led a most abandoned life, and who was apparently in a dying state from extreme exhaustion. Her age might be about thirty-four. On inquiry, I learnt that for the two preceding years she had suffered from repeated and severe hæmorrhages from the uterus, and that, on the present occasion, the discharge had continued for many days, until, when I saw her, though still abundant in quantity, it was not more coloured than the serum of healthy blood. In short, she seemed to be rapidly sinking. I ordered her immediately a full dose of the acetate of lead, with opium, to be repeated every four hours; and Port wine or brandy, in sago, to be given frequently. Cold was applied, both externally and internally, by means of an injection of a strong solution of alum. For a brief space of time she appeared somewhat relieved; but in two or three days the hæmorrhage recurred with increased violence, the fluid also being more sanguineous. Having, in other cases, fancied that the ergot had been of use, I immediately gave her ʒss. of the powder, to be repeated in ℈i. doses every three or four hours. From the lapse of an hour after the first dose she experienced manifest relief; and on seeing her in the evening, I directed the third dose not to be given till the sixth hour, unless any recurrence of the discharge should render it necessary. Next day she continued better; and the medicine was directed to be taken only night and morning. From this visit she went on gradually improving; and for two years after, when I had occasion to prescribe for her, she had experienced no return of hæmorrhage. She appeared quite well in health, but completely blanched in colour. To this I might add several other cases, occurring in persons of asthenic constitution, in which similar good effects were derived from the ergot, but consider that it would only be a needless occupation of your columns, and your readers' time. It may be enough to say, that seven years of subsequent practice have confirmed my reliance on the ergot in such cases. Even where an opposite diathesis prevails it has been employed advantageously, combined with conium and hyoscyamus. In such persons it is advisable to premise a moderate abstraction of blood from the system, and to keep up a depletory action by means of saline cathartics. In these cases, however, the infusion of roses with sulphuric acid and digitalis, or alum, seem more appropriate.

In leucorrhœa it has been highly useful. In this disease, as in menorrhagia, it is necessary to ascertain the state of the system, more particularly the uterine disorder on which it depends; as where any degree of inflammation is present it will be injurious. In several distressing cases of this disease, where the strongest astringent injections had been employed without any effect, except exciting inflammatory action which did not previously exist, I have found the ergot, aided by injections of simple warm water, or the decoction of poppy capsules, perfectly successful. I may observe *en passant* that from considerable opportunities of observation, it is my firm belief that in a vast proportion of cases of leucorrhœa stimulant and astringent injections are not only uncalled for, but absolutely contraindicted; at the same time, it must be acknowledged that they are occasionally of great use.

In chlorosis and amenorrhœa I have frequently experienced the good effects of the ergot, after aloes, iron, valerian, cantharides, &c., had all been employed without the slightest advantage. In these cases, when extreme nervous susceptibility exists, it may be most advantageously combined with the valerian, and where the alvine system is torpid, with aloes. When aloes are employed, I attach the preference to the Barbadoes: in doing so I may be somewhat empirical, as it is difficult to account for the superiority of the Barbadoes over the socotrine, as an emenagogue, unless it be ascribed to the larger quantity of bitter principle contained in the former.

In dysmenorrhœa the ergot has been most useful. In one case, accompanied with what may without exaggeration be termed torture at each period, it appeared almost magical in its operation. It is right to state that it was given in combination with the valerian; which medicine, however, had been previously given, but without any apparent benefit. To enter into the detail of individual cases is not only tedious, but would also occupy too much of your valuable columns. I shall therefore endeavour, with the utmost possible brevity, to explain the apparent paradox of a medicine being of use in cases at first sight so pathologically opposite.

It is well known that the same cause operating on different constitutions will be followed by effects proportionately variable. This is a fact which does not admit of refutation, whether it be applied to man morally or physically. So then it is with regard to the uterine system, as every man of moderate experience will admit that the same morbid condition will, according to peculiarity of constitution, produce a very dissimilar train of symptoms. Thus, whilst a congested state of the uterine vessels will in one person lead to menorrhagia, the same cause operating on a different constitution will be attended with leucorrhœa.—Again, when the nervous system of the uterus is at fault, we in one person have chorea, in another simple hysteria, in others cardiac and pulmonic symptoms, &c. &c. This is especially true in regard to menorrhagia and leucorrhœa. These diseases may arise alike in the sthenic and asthenic diatheses, which must of course render a corresponding modification of our remedial measures necessary. Without using much discrimination, we aggravate rather than alleviate the suffering inseparable from disease, and enrol ourselves under the banner of empiricism.

I have merely to add, that I consider myself fully justified in recommending the ergot as an excellent emenagogue and anti-hæmorrhagic agent. Should this recommendation be followed by beneficial results in the hands of other professional men, I will regard myself as amply rewarded for the trouble of putting together the foregoing remarks, the correctness of which can only be proved by trials more extensive than private practice; besides which, every man is liable to view with *partiality* any plan of treatment, which, without authority, he adopts and finds successful.

P. S. The doses in which I have prescribed it, have varied from gr. x. to ℈j. of the powder, ʒss. to ʒj. of the concentrated tincture. A good formula for decoction is given in Foot's Medical Almanack, although it appears to me that the quantity of ergot is rather small.

My friend Mr. Bennet, of Gateshead, informs me that he has given the ergot in a case of hæmoptysis with very satisfactory results.—*London Med. Gaz.*

---

*On Luscitas, with some Observations on the Pathology of the Ophthalmic Muscles, and on some other points connected with Strabismus.* By Aug. Franz, M. D. Leipsic, M. R. C. S., &c.—Since medical men have been seized with the monomania for squint cutting, much has been written on strabismus, but little or nothing, to my knowledge, on luscitas. As I have lately met with a case of luscitas spatica, and found in the last number of von Ammon's Monatsschrift for 1840, p. 550, the observation made by Dr. Rigler, that luscitas never admits of operation, and that Dr. Melchoir, in his "Dissertatio de Myotomia Oculi," [Hauniæ, 1841, mense Martio,] p. 21, regards an operation in these cases in the light of an experiment, I felt induced to give this subject a little closer consideration, and more especially with respect to treatment by operation.

Although the term luscitas has been applied by authors to a variety of ophthalmic affections, Prof. Beer restricts it to those cases where the eye is turned, as in strabismus, to the one or other side, but cannot be moved at the will of the patient; when the sound eye is closed, the eye affected remains completely fixed in an abnormal position.

As the principal causes of this affection, may be considered a deficient or faulty formation of the orbit, the eye-ball, its muscles, or of the eyelids; enlargement of the lachrymal gland, adventitious growths or abscesses in the orbit; symblepharon affecting a squinting eye to a great extent; idiopathic or metastatic inflammations of the eye or its appendages; strophia, malacia, paralysis, hypertrophy, or spasm of one or more muscles.

If a deficient or faulty formation of the orbit, the muscles, &c., is the cause of luscitas, it is evident that little or nothing can be done for its relief; if enlargement of the lachrymal gland, or adventitious growths in the orbit, these diseases must be treated by pharmaceutical means, or operation, according to the indications in the case. An abscess in the orbit must of course be opened as early as possible, in order to prevent sinuses or accumulations of matter, which in this situation would create great mischief. If symblepharon exists in a squinting eye, the union of the lid with the ball must first be separated, by which proceeding the case is reduced to simple strabismus, and is then to be operated on in the usual manner. A case of this kind came under my treatment, and was cured on this plan.

Atrophy of one or more muscles of the eye may be occasioned by want of exercise, when the eye has been affected with strabismus for a long time, and has not been used on this account; or by insufficient supply of blood to the muscles in consequence of disease, injury, pressure on the arteries, &c.; perhaps also by ossification of the arteries given off to the muscles, just as we find the arteria centralis ossified; or by suppuration being the sequel of a vehement inflammation. In these cases the nutritive functions are diminished, but they may also be deranged, for instance, after chronic blenorrhœa, and then malacia, or softening of the muscle may take place. In both these instances the muscle loses its normal irritability, but more especially its power of contraction, and then becomes atonic and lax. The same diminution or total loss of muscular tone follows, when the innervation of the nerves of motion is entirely or partially suspended, as in a paralytic state of the muscle from rheumatism, or from apoplexy, pressure on or softening of the brain, &c. Now when a muscle is affected with the maladies just mentioned, it is more or less unable to counteract the ordinary action of its healthy opponent, in which therefore the contractile power is permitted to gain the preponderance, and in time an actual or habitual contraction is established. From the diminution of power on the one side, and excess of it on the other, the eyeball has of course lost its proper poiition in the orbit, and is turned towards the contracted muscle, from which position it can only be slightly moved, or in which it is completely fixed.

As astrophy and malacia frequently come on slowly, and last for a long time, until the eyeball becomes absolutely fixed in an abnormal position, or luscitas is established, the time is entirely lost where the treatment by pharmaceutical means might have been advisable; and, in my opinion, an operation should be performed under such circumstances, provided the weakened muscle is not completely paralysed or otherwise totally disorganised—a circumstance certainly not easily ascertained beforehand. If in such cases the muscle which draws the eye out of its proper position is divided, and all additional impediments are removed which might tend to limit the motion of the eye, the weakened muscle is placed in a position more favourable for acquiring its lost power by exercise, as also for the action of local remedies upon the muscle, since it is no longer kept in a state of continued extension. But as an operation in these cases has only been the first step towards a cure, the ophthalmic orthopedie becomes now of the greatest importance. The healthy eye must therefore be bound up with a light bandage during the whole day for the first fortnight. After this time this plan must be persevered in at intervals for several weeks. While the eye is bound up a small shade must be fastened before the operated eye, in such a manner that the patient is forced to turn his eye in a direction exactly opposite to that it held before the operation, to be enabled to see. He must moreover be ordered to fix his eye firmly on a distant object. I have pursued this plan in several cases of inversion dependent on a paralytic state of the external rectus. The most severe case of this kind which came under my care, and which I beg to relate more fully, occurred in a young gentleman from Staffordshire, recommended to me by Dr. Ingleby of Birmingham.

H. L., aged 9, of strumous habit, was born with his eyes perfectly straight, but three months after birth both were observed to turn in, and in time became more inverted. This double inversion appeared to be owing to an early hydrocephalus, as I was led to believe by circumstances elicited by a strict examination, and by the whole formation of the head. When I for the first time saw this patient, both eyes were inverted to such an extent that only about five-sixths of the right and two-thirds of the left cornea could be seen. The pupil of the right was normal in size and action, but the left rather contracted and slow in action. In ordinary or inattentive vision, both eyes remained in their usual inverted position; the patient was in the habit of constantly moving the body laterally, and the head in a semi-rotatory or circular manner, by which complicated movements of head and body (endeavours to compensate for the limited motion of the eyes) all objects presented themselves to the right eye, on account of this being in a more favourable position than the other, but were of course indistinctly seen. If now any object struck his attention, he kept his eye upon it, and turned the head so as to bring the pupil, which could be moved from the internal canthus a little beyond the centre of the orbit, into its proper position; keeping then the head stationary, he saw even small objects very distinctly; but as soon as his attention was distracted the eye resumed its usual abnormal position. With the left eye, which could be moved but slightly and with difficulty when the other was closed, the patient saw objects almost as well as with the right, when the head, eye, and object were brought in such a position that the visual axis was in a line with the object viewed. The patient used only one eye for seeing, generally the right, leaving the other entirely inactive, on which account no double vision was present.

On the 2d of July, 1840, I divided the internal rectus on the left side, which produced no effect in the position of the eye or in facilitating its motion outwards. I now proceeded to separate the cellular tissue between the muscle and the ball, extended the wound in the conjunctiva, and divided the inner border of the superior and inferior rectus, but also without effect, and at last divided the superior oblique, after which proceeding the eye certainly would be turned further outwards, and with greater facility, although it could not be kept in this position, as on cessation of the will the inversion was as great as before the operation. Having thus freed the eye from all mechanical influence which tended to keep it inverted, the right eye was bound up, and a shade fastened before the left, so as to oblige the patient to turn the eye as much as possible outwards in order to be enabled to see; he was moreover ordered to fix the eye thus shaded frequently on a distant object. Ung. Hydr. Mit. was rubbed into the neighbourhood of the inner canthus, to retard the healing of the muscle. An ointment composed of Phosphorus dissolved in Ol. Rosmar. and Bals. Peruv. was rubbed around the external canthus and the temples of both eyes. Ung. Ant. Pot. Tart. was applied to the vertex, and the bowels were regularly kept open. A week after the operation the ophthalmic fountain (described Med. Gaz. vol. xxvii. p. 444,) was used. This treatment was continued until the patient was again brought to town, when I found the left eye not half as much inverted as before the operation, and that both eyes could be turned outwards beyond the orbital axis.

On the 22d of August the rectus internus of the right eye was divided, and the superior and inferior rectus partially. Immediately after this operation the eye assumed its proper position for a few minutes, after which it was again somewhat inverted. By closing the left eye and fastening the shade before the right, in the manner above described, the external muscle of this eye was chiefly exercised up to the 26th of August, when the rectus internus of the left eye was again divided, after which operation both eyes daily improved by judicious use, so that when the patient left the town on the 31st of August, the right eye was perfectly straight, and the left nearly so. In walking, the carriage was more steady, and the movements of the head diminished and less awkward. He was ordered to look frequently with both eyes at distant objects (as in shooting at a target) to exercise principally the external rectus of the left eye, to continue the strengthening ointment to the temple and lids on both sides, as also the ophthalmic fountain, to go to the sea-side, and to take frequent exercise in the open air.

The mother, who bestowed the greatest care upon the patient, he being an only child, wrote to me from time to time that the left eye slowly improved in position, and that both could be freely moved towards the external canthus, and in her last letter of the 20th of March, 1841, she observes, "My little son's eyes are now perfectly straight; there has not appeared the least inversion for I should think the last two months; before that time fatigue or change of weather considerably affected them, but such is not the case now. They have been examined by several professional men, who are unanimous in the opinion of its being a perfect cure. He can turn the eyes out much further than the generality of persons can."

There still remains another class of causes for consideration which may produce luscitas. The circumstance of three distinct cerebral nerves being distributed exclusively to the six comparatively small muscles of the eyes accounts sufficiently for the great predisposition to nervous excitement and spasmodic affections. Certain injuries and inflammations of the eye, irritation of the brain from mental or organic causes during dentition, or reflected from more distant parts of the body, as the abdominal organs, or from the peripheric nervous system, as *e. g.* after scarlatina, &c. frequently give rise to convulsions of the ophthalmic muscles. The local irritation accompanying these convulsions augments the muscular irritability, but chiefly the contractile power, and in time produces some change of structure in the muscle while contracted, the result of which is absolute and permanent shortening. Together with this increase of muscular irritability, the innervation or the action of the arteries may be augmented—conditions on which hypertrophy of the muscle depends, which I have principally met with when the strabismus was preceded by inflammations of an acute character, directly affecting the eye, or its muscles, but which I think may also be caused in cases where inflammatory affections never existed, merely by too copious an afflux of blood to the muscle. If the convulsions have been caused by a purely nervous irritation reflected from distant parts of the body, *e. g.* in worms, I have found the muscle rather thin, hard, and more tendinous. After protracted ophthalmia, which begins with great vehemence, and is the effect of an exanthematic or rheumatic metastasis, the innervation may gradually become diminished or the nutritive functions impaired, so that paralysis or atrophy may be added to structural shortening, which originated with metastasis at the commencement of the inflammation.

Under all these circumstances the eyeball is turned towards the shortened muscle, and in time becomes permanently and more or less immoveably fixed in an abnormal position; in the same ratio as the shortened muscle, from change of structure, loses its elasticity, and its antagonist its energy, from being in a constant state of extension. Luscitas, or strabismus from convulsions of the muscles, is, therefore, generally slowly established; but it may also occur suddenly after hysteria, hypochondria, epilepsy, violent mental emotions, or external agents acting chemically or mechanically on the eyeball. I once saw a case, under Prof. F. Jaeger, of Vienna, where an hypochondriac, after some errors of diet and mental excitement during dinner, was suddenly affected with a complete luscitas of his left eye. As this person sought medical advice directly, the complete immoveability of the eye soon entirely ceased after the administration of an emetic and some other remedies, which rectify the functions of the digestive organs.

I am of opinion that, with few exceptions, almost every recent case of luscitas or strabismus, and even several of some time standing, which have been brought on by a spasmodic state of the muscle, may be cured by pharmaceutical means, provided the real cause of the spasm can be detected, and proper means are employed. From my own observations it appears to me, that in all recent cases of this character, whatever plan of treatment the remote cause may require, those means which are locally applied should be antiphlogistic or antispasmodic, as no doubt can exist that stimulating applications will increase the mischief. I have attended several cases of squinting of this kind, brought on by dentition, worms, or disorders of the stomach, and irritation of the brain, where I succeeded in accomplishing a cure by therapeutic means; and in a late number of the Medical Gazette, I have related a case of complete luscitas, caused by spasm from an acute rheumatic attck, which was cured in a short time without operation. Some time ago a lady asked my advice for a slight degree of strabismus affecting each eye alternately, and being of eight years' duration. Whlle speaking with her, I observed the left eye affected when she was looking at me, and the right when she looked at a more distant object; I therefore concluded that in this case the strabismus might be dependent on an inequality of the power of vision of the two eyes, and on some irregular action of the muscles, and, on close examination, found a considerable degree of myopia in the right eye, and a rather presbyopic state of the left. In looking at a near object she was in the habit of using the right eye; and in viewing distant objects she used the left, leaving in each case the other eye to the casual action of its muscles, which always drew it slightly outwards. This case of alternating strabismus was cured by local bleeding of the right eye, the use of gentle aperients, by binding up the left while the myopodiorthoticon was employed to the right eye.

If proper local pharmaceutical means have been employed for some time without effect, and if the remote cause has been removed, or at least has ceased to act on the ophthalmic muscles, or if the luscitas or strabismus has lasted for a considerable length of time, I think we may firmly conclude that the spasmodic contraction of the muscles has become habitual, or that structural shortening is already established, which now remains as a secondary effect, and therefore, even if the case is a complete luscitas, this cannot be considered as a contra-indication to the performance of an operation. As the muscle is already absolutely shortened, I think it not advisable to divide it in its muscular fibres, much less to excise a portion, as some do, or to push the posterior part back into the orbit, as by these means the muscle would be made still shorter; but it is more proper to divide it in its tendinous portion close to the sclerotica, so that the muscle may retain its whole length, and thus be allowed to attach itself to the sclerotica as far anteriorly as possible, since then the movements of the eyeball towards the divided muscle will be less limited. If, after the tendon and all additional frænula have been completely cut across, the abnormal position is still considerable, this will generally be remedied either by separating the cellular tissue between the muscle and the globe more or less, or by enlarging the wound in the conjunctiva (especially if this membrane is thickened, tough, or inelastic,) or by partial division of the inner border of the superior or inferior rectus, as the case requires; but in all these steps the greatest care must be taken, especially if the opposite muscle acts powerfully, to cut rather too little than too much, as otherwise the strabismus will not be cured, but squinting towards the opposite side will be substituted for it. I have never operated on the muscles of a sound eye in order to set its fellow straight; but in cases of double strabismus, if the one eye was not rendered quite straight by a simple division of the muscle, I at once proceeded to operate upon the other eye, which plan was generally attended with success without the additional steps above related. The first of these plans should be pursued in all cases where the antagonist of the divided muscle is more or less paralyzed, since it then has a better chance of regaining its tone by free exercise. If, after these proceedings, in cases of inversion, the eye remains still in an abnormal position to a great extent, I divide the superior oblique.

Although we do not even yet perfectly understand the action of the obliques, I think it will be admitted that if either or both are shortened in structure, which they may be as well as the recti, because both groups of muscles are subject to the same pathological conditions, they will increase the inversion; and that if after the rectus internus, and all other attachments which may retain the eye in its unnatural position, have carefully been cut, some degree of inversion still continues, these muscles, but principally the superior oblique, may be the cause of it, since this, (being the longest of the six) is subject to become more shortened than the rest. This I have found to be the case in several instances. I do not mean to say that we should thoughtlessly cut across the superior oblique in all obstinate cases of inversion, since by the proceedings above detailed we frequently succeed in removing the affection completely; but where all these proceedings have had no satisfactory effect, I am convinced that a division of this muscle will either rectify the position of the eye directly, or when some degree of paralysis of the external rectus is the cause of the continuation of the inversion, and if then in such cases the shortened superior oblique is an impediment to the motion of the eyeball towards the external canthus, its division will remove this impediment, and thus the external rectus will be placed in a situation far more favourable for the recovery of its proper action by free exercise.—*Ibid.*

---

*Successful Case of Extirpation of the Neck of the Uterus.* By M. Francesco Rizzoli of Bologna.—A woman, 25 years of age, was admitted into the hospital at Bologna on the 17th July, 18s9, on account of disease of the uterus. She had been for several years labouring under symptoms of virulent gonorrhœa, and still suffered from it. The neck of the uterus was found enlarged, hardened, and ulcerated, but it was impossible to decide whether the alteration in the structure depended on a syphilitic cause or on cancer. She was therefore for the space of a month put on the anti-syphilitic regimen, but as the uterine affection continued to advance, and was not in the least benefitted by it, it was decided to remove it by operation. The uterus was therefore seized with forceps, and gradually pulled down, so as to bring its mouth and neck to the level of the labia, when the diseased parts were removed with a curved bistoury. The hæmorrhage which followed the operation was slight. No unpleasant symptoms resulted; the wound soon cicatrized; and by the twentieth day after the operation, the cure was completed. The portion which was removed presented the true scirrhous structure.—*Ed. Med and Surg. Journ.*, *from Bulletino delle Scienze Med.*, September, 1840.

---

*Facts relative to the Statistics of Menstruation.* By Dr. Adelmann of Fulda.—During the years 1834, 35, and 36, Dr. Adlemann ascertained the period at which menstruation had commenced in 507 individuals. From this it appeared that in girls with black hair, the average age at which menstruation commenced was 16; in girls with brown hair, the average age was 17; and in girls with fair hair, between 16 and 17. The average duration of each menstrual period, four to five days for the black-haired girls, and three to four days for the brown and fair-haired. Only one in 102 cases was met with who menstruated regularly at the interval of three weeks; all the rest did so at regular periods of four weeks.—*Ibid.*, *from Neue Zeitschrift fur Geburtskunde.* August, 1840.

